# Is the Use of Tourniquets More Advantageous than Other Bleeding Control Techniques in Patients with Limb Hemorrhage? A Systematic Review and Meta-Analysis

**DOI:** 10.3390/medicina61010093

**Published:** 2025-01-09

**Authors:** Roberto Cirocchi, Dominica Prigorschi, Luca Properzi, Matteo Matteucci, Francesca Duro, Giovanni Domenico Tebala, Bruno Cirillo, Paolo Sapienza, Gioia Brachini, Sara Lauricella, Diletta Cassini, Antonia Rizzuto, Andrea Mingoli

**Affiliations:** 1 Department of Surgery, General Surgery, University of Perugia, 06123 Perugia, Italy; roberto.cirocchi@unipg.it (R.C.); dominica.prigorschi@studenti.unipg.it (D.P.); durofrancesca3@gmail.com (F.D.); 2 Department of Surgery, General Surgery, University of Milan, 20122 Milan, Italy; matteo.matteucci@unimi.it; 3 Azienda Ospedaliera S. Maria, 05100 Terni, Italy; g.tebala@aospterni.it; 4 Department of Surgery, General Surgery, Sapienza University of Rome, 00185 Roma, Italy; bruno.cirillo@uniroma1.it (B.C.); gioia.brachini@uniroma1.it (G.B.); paolo.sapienza@uniroma1.it (P.S.); andrea.mingoli@uniroma1.it (A.M.); 5 Fondazione IRCCS Istituto Nazionale Dei Tumori, Department of Surgery, Colorectal Surgery Division, 20133 Milan, Italy; lauricella3008@gmail.com; 6 ASST Nord Milano-Department of General and Rery, Sesto San Giovanni Hospital, 20099 Sesto San Giovanni, Italy; diletta.cassini@asst-nordmilano.it; 7 Department of Medical and Surgical Sciences, University Magna Græcia of Catanzaro, 88100 Catanzaro, Italy; arizzuto@unicz.it

**Keywords:** tourniquet, trauma, amputation, bleed

## Abstract

*Background and Objectives:* Trauma, particularly uncontrolled bleeding, is a major cause of death. Recent evidence-based guidelines recommend the use of a tourniquet when life-threating limb bleeding cannot be controlled with direct pressure. Prehospital hemorrhage management, according to the XABCDE protocol, emphasizes the critical role of tourniquets in controlling massive bleeding. The aim of this systematic review and meta-analysis was to summarize data from the available scientific literature on the effectiveness of prehospital tourniquet use for extremity bleeding. *Materials and Methods:* A systematic review and meta-analysis was performed between March 2022 and March 2024, adhering to PRISMA guidelines, to determine whether prehospital tourniquets are clinically effective. The protocol was published on PROSPERO (ID number: CRD42023450373). *Results:* A comprehensive literature search yielded 925 articles and 11 studies meeting the inclusion criteria. The analysis showed a non-statistically significant reduction in mortality risk with tourniquet application (4.02% vs. 6.43%, RR 0.70, 95% CI 0.46–1.07). Analysis of outcomes of amputation of the traumatized limb indicated a statistically higher incidence of initial amputation in the tourniquet group (19.32% vs. 6.4%, RR 2.07, 95% CI 1.21–3.52), while delayed amputation showed no difference (9.39% vs. 3.66%, RR 0.93, 95% CI 0.42–2.07). Tourniquet use demonstrated a non-significant reduction in the number of blood components transfused (MD = −0.65; 95% CI −5.23 to 3.93 for pRBC, MD = −0.55; 95% CI −4.06 to 2.97 for plasma). *Conclusions:* Despite increasing use in civilian settings, this systematic review and meta-analysis showed no significant reduction in mortality or blood product use associated with prehospital tourniquet use. Further research, including high-quality randomized controlled trials, is required, as well as awareness and education campaigns relating to proper tourniquet use in the prehospital setting.

## 1. Background

The main causes of injury deaths worldwide are road injuries (29%), self-harm (16%), falls (15%), other unintentional injuries (14%), interpersonal violence (11%), drowning (5%), fire, heat and hot substances (3%), exposure to mechanical forces (3%), poisonings (2%), collective violence, and legal intervention (2%) [1].

Uncontrolled bleeding is one of the leading preventable causes of death in trauma patients. The loss of 30–40% of total blood volume can result in hypovolemic shock, which, without prompt intervention, may lead to death. This information emphasizes the urgency of effective prehospital bleeding control techniques such as tourniquets [2].

Prehospital management of bleeding is a key stage in the management of trauma patients, and it corresponds to stage C of the ABCDE approach recommended by ATLS (Advanced Trauma and Life Support). In 2018, Prehospital Trauma Life Support (PHTLS) changed the ABCDE protocol to XABCDE, where X stands for “exsanguinating”, indicating the priority of managing massive bleeding before assessing the airway [3].

Bleeding control techniques include direct pressure, hemostatic dressings for junctional bleedings, and tourniquet application for extremity bleedings.

The manual pressure points (MPP) technique has been described in military first aid manuals as early as 1988. The general premise of the technique is occlusion of distal blood flow, which is achieved by applying pressure on the arterial supply. Therefore, it offers an alternative means of potential hemorrhage control, without the requirement of any dedicated or improvised equipment. Although the technique has been described and presumably used for several decades, evidence of its applicability in and efficacy in real-world settings has remained scarce [4].

The last decade has witnessed a surge of products designed to manage severe bleeding. Two primary factors affect the efficacy of hemostatic products. The first is the mechanism of action. Some agents concentrate clotting factors at the site of injury, others form a mucoadhesive seal around the wound, and yet others are procoagulants that either activate the coagulation cascade or provide exogenous clotting factors to the injury site. The second factor affecting efficacy is the form in which the agent is delivered: wafer, granule, or gauze. The ability to conform to the geometry of the wound is essential for the efficacy of the agent [4].

Tourniquets, as a key prehospital intervention, have been shown to be life-saving devices when used correctly [4]. By controlling bleeding at the earliest possible stage, tourniquets not only save lives, but also stabilize patients, improving their chances of survival upon arrival at medical facilities [5].

The tourniquet is a device that is placed around a bleeding limb and works by creating a pressure on the limbs that closes the arteries and stops blood from flowing. It must be applied at least 5 cm from the proximal edge of the wound and tightened until bleeding stops and the distal pulse disappears. Tourniquets have been shown to be safe when used for less than 2 h [4].

Complications may result from improper, unnecessary, or prolonged tourniquet application, including paradoxical bleeding (due to inadequate tourniquet tightness, in some cases, insufficient pressure may compress the veins but not the arteries, leading to an increase in venous pressure in the area of the hemorrhage, which can even worsen the bleeding), ischemia, limb loss, and nerve and soft tissue injuries [4].

The effectiveness and safety of tourniquets have been proven in military studies [5,6,7]. Tourniquet application has become one of the primary first aid maneuvers in the military setting, and all military personnel, not just doctors, are equipped with tourniquets and trained in their use.

In the civilian setting, however, the adoption of tourniquets has been slowed by a number of factors, such as differences between civilian and military injuries (explosions and high-energy penetrating injuries in the military context vs. injuries with a contusion mechanism in the civilian context), different demographic characteristics of patients, faster access to primary care in the civilian context, and the risk of complications from prolonged or unnecessary tourniquet application [8,9,10,11].

Despite their proven efficacy in the military context, and the recommendations of leading organizations, the use of tourniquets in the civilian context is not yet homogeneously widespread. The aim of this systematic review and meta-analysis is to evaluate the clinical effectiveness of prehospital tourniquets for controlling extremity bleeding, with a specific focus on mortality reduction, limb salvage, and transfusion requirements in both civilian and military settings.

## 2. Methods

Our systematic review and meta-analysis were conducted according to the Preferred Reporting Items for Systematic Reviews and Meta-Analyses (PRISMA) guidelines [12]. The protocol was published on PROSPERO (ID number: CRD42023450373). The research question was as follows: “Are prehospital tourniquets clinically effective for extremity bleeding control?”.

### 2.1. Search Strategy

The systematic literature search, which started in March 2022 and ended on 31 March 2024, was conducted on PubMed, SCOPUS, and Web of Science (WOS). We included studies from 2008 to 2024. On PubMed, the terms used for the search were “tourniquet and trauma and prehospital”. Two search enhancements were used through the use of the “related articles” feature of PubMed and the use of Google Scholar. No language restrictions were applied.

### 2.2. Selection Criteria

Two different investigators screened the studies. Any disagreement was resolved by mutual agreement. Observational studies and randomized controlled trials were eligible for inclusion. Case reports, editorials, letters, reviews, and studies with irrelevant or incomplete data were excluded. Reference lists were searched for additional relevant articles. To identify all relevant studies, the following inclusion criteria were used: (1) population: patients of any age with severe bleeding from an extremity wound; (2) intervention: comparison of the emergency treatment of external hemorrhage (pneumatic/mechanical tourniquet vs. no tourniquet application); (3) setting: prehospital setting; and (4) outcomes: mortality, infused blood components, or amputation of the traumatized limb.

### 2.3. Study Selection and Data Extraction

The following information was extracted from each study by D.P. and R.C.: first author’s last name and year of publication, country of the hospital in which the study was conducted, type of study, sample size, trauma mechanism, patients’ characteristics, intervention type, and outcomes. An attempt was made to contact the corresponding authors of the studies included in the meta-analysis to request the necessary information. However, responses were not consistently received, and this remains a limitation of the study.

### 2.4. Outcomes

The outcome measures selected for the analysis were as follows: (1) in-hospital mortality; (2) infused blood components (erythrocytes, plasma); and (3) amputation of the injured limb at the time of hospital admission and late amputation.

### 2.5. Data Synthesis

In dichotomous data analysis, we used risk ratio (RR) with 95% confidence intervals (CIs); in contrast, for continuous outcomes, we used mean difference (MD) with 95% CIs. We identified heterogeneity (inconsistency) by visually inspecting forest plots and by using a standard Chi^2^ test with a significance level of 75%. We used RevMan 5 as the analysis program for the meta-analysis.

### 2.6. Quality of Evidence

The risk of bias was evaluated by two independent investigators using the ROBINS-I tool.

## 3. Results

**Figure 1:**
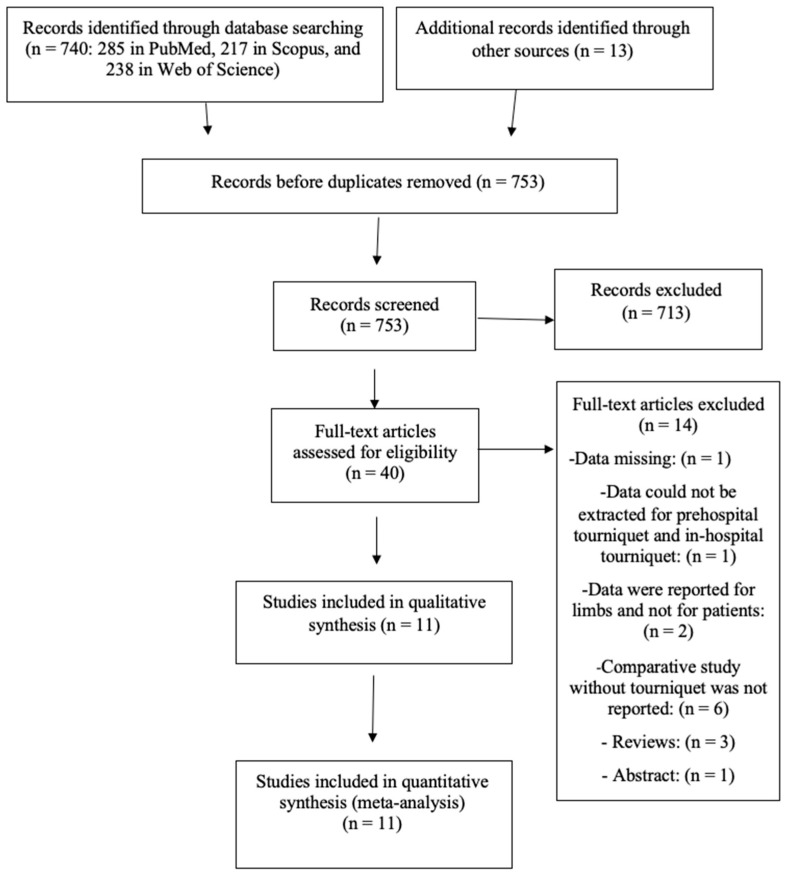
Prisma Flow Chart.

The study selection process is shown in Figure 1; in the searches, 925 articles were screened, 14 full texts were excluded, and 11 studies were included in quantitative synthesis (meta-analysis).

The 14 excluded studies were excluded for the following reasons:

In one paper, data about tourniquets were missing [13]; in another paper, data could not be extracted for prehospital tourniquets and in-hospital tourniquet [14]; in two studies, data were reported for limbs and not for patients [8,15]; in six studies, a comparative study without a tourniquet was not reported [16,17,18,19,20,21]; three studies were reviews [22,23,24]; and one study was an abstract [25].

Of the 11 included studies, 10 [11,26,27,28,29,30,31,32,33,34] were retrospective observational studies, and 1 [8] was a prospective observational study.

The studies reviewed were conducted in the United States of America [26,27,28,29,30,31,32], Canada [33], Iraq, and Afghanistan [8,11,34], with data collected from trauma registries, medical records and databases. Four of the studies [8,30,34] were conducted in military settings and seven in civilian settings [26,27,28,30,31,32,33].

All the 11 studies compared the control of external limb bleeding with prehospital tourniquet application vs. no prehospital tourniquet application. A total of 3686 patients were included. Table 1 shows the main characteristics of the studies and patients.

The prehospital vital signs of patients reported in the 11 studies are shown in Supplementary Table S1. Injury Severity Score (ISS) was generally comparable across all studies between the tourniquet and non-tourniquet groups, with only slight variations depending on the study. The specific vessels involved in the injuries were reported in only three of the studies [27,28,31], which limits broad generalization; however, these primarily involved the extremity arterial vessels (Supplementary Table S2). The mechanism of injury was reported in seven studies [26,27,28,29,31,32,34], and varied between civilian and military settings, with blunt trauma predominant in civilian cases and penetrating injuries more common in military scenarios (Supplementary Table S3). A brief summary of the data is presented in Supplementary Tables S1–S3. Supplementary Table S1: across all the studies, prehospital vital signs were documented. The Injury Severity Score (ISS) was generally comparable between the tourniquet and non-tourniquet groups, with slight variations depending on the study. Supplementary Table S2: the list of vascular injuries, as provided in the studies, primarily involved extremity arterial injuries. However, these data were only reported in a subset of studies, which limits broader generalization. Supplementary Table S3: the mechanisms of injury varied between civilian and military settings, with blunt trauma predominant in civilian cases and penetrating injuries being more common in military scenarios.

We conducted a subgroup analysis for the studies that were conducted in a civilian setting and those that were conducted in a military setting.

The marked discrepancy in male sex proportion in the TQ-group in the study by Smith (2019), compared to the other studies, is notable. This could be attributed to demographic or selection biases inherent to the study population, or regional variations in trauma incidence and management. For instance, the study by Smith (2019) primarily involved civilian settings, which may differ in terms of mechanisms of injury and prehospital practices compared to studies conducted in military or mixed populations. This heterogeneity underscores the need for cautious interpretation of demographic variables across studies [29].

### 3.1. Results of Quality of Evidence

**Figure 2:**
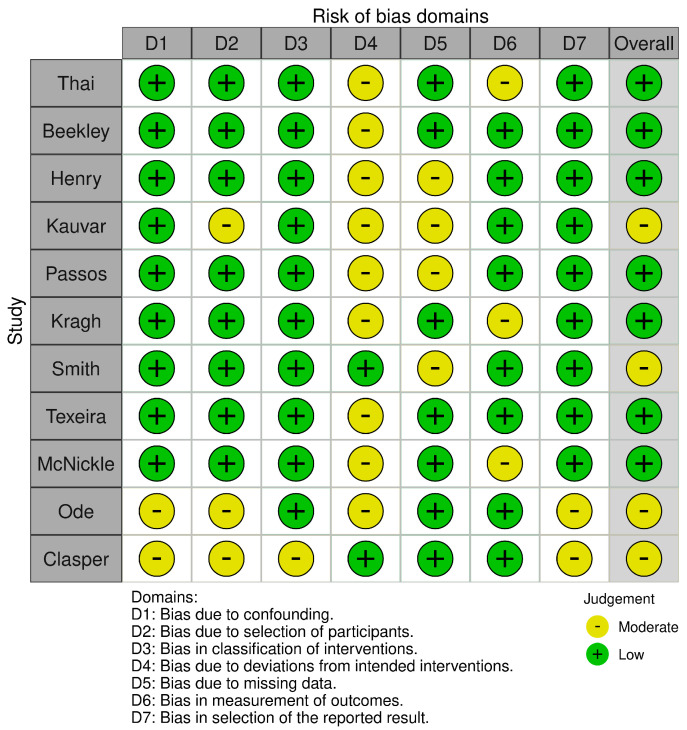
Risk of bias according to Robins-I tool [9,11,26,27,28,29,30,31,32,33,34].

**Figure 3:**
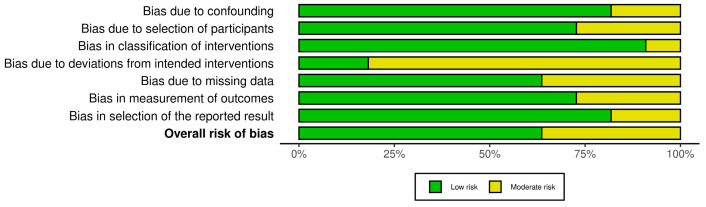
Risk of bias according to Robins-I tool.

The ROBINS-I tool probes for potential risk of bias in seven study domains (bias due to confounding, bias in the selection of participants for the study, bias in the classification of interventions, bias due to deviations from intended interventions, bias due to missing data, bias in the measurement of outcomes, and bias in the selection of the reported result). The risk of bias within each domain is normally categorized as “Low”, “High” or “Moderate”. Nine of the studies included showed a low risk of bias in domain one (bias due to confounding) [8,11,26,27,28,29,30,31,33] and ten showed a low risk of bias in domain three (bias in classification of interventions) [8,11,26,27,28,29,30,31,32,33], while two studies presented a moderate risk of bias in these domains [32,34], in domain one and [34] domain three. The risk was low for eight of the studies in the second domain (bias in the selection of participants for the study) [8,11,26,27,28,29,31,33], while three studies presented a moderate risk in this domain [30,32,34]. Nine studies showed a moderate risk of bias in domain four (bias due to deviations from interventions) [8,26,27,28,30,31,32,33,34] and four studies showed a moderate risk in domain five (bias due to missing data) [27,29,30,33], while the risk of bias in these domains was low for the other studies included in this review. The bias in the measurement of outcomes was moderate for three studies [8,26,28], while it was low for the other studies. At least nine of studies included showed a low risk of bias in domain seven (bias in the selection of the reported result). Two studies [32,34] showed a moderate risk of bias in this domain (Figure 2 and Figure 3).

### 3.2. Primary Outcomes

#### 3.2.1. In-Hospital Mortality

Overall, nine observational studies (3410 patients) analyzed this outcome [11,27,28,29,30,31,32,33]. Mortality at 24 h, 30 days, or 12 months was never reported, nor was the cause of death.

**Figure 4:**
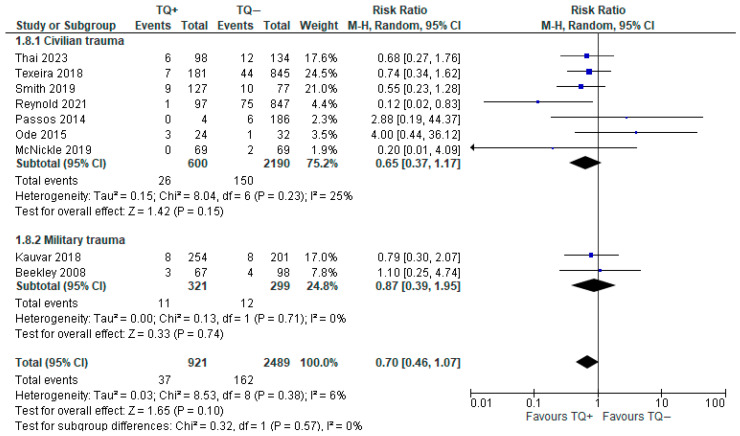
Risk ratio for mortality with prehospital TQ (TQ+) vs. no prehospital TQ (TQ−) [26,27,28,29,30,31,32,33,34].

Despite the differences among the included observational studies, the forest plot showed a slightly lower risk of mortality with prehospital tourniquet application (4.02%), compared with the no tourniquet group (6.43%); however, this result was not statistically significant (RR 0.70, 95% CI 0.70–1.07) (Figure 4). Heterogeneity was found to be low (I^2^ = 6%). The same trend was reported in the results of the subgroup analysis performed for the military and civilian traumatized patients (Figure 4).

The results presented in Figure 4 show no significant reduction in mortality associated with tourniquet use in military studies, despite the Background section highlighting their established efficacy in such settings. This discrepancy might be explained by methodological differences, such as study design, patient selection, or outcomes reported. Additionally, the heterogeneity in military injuries (e.g., severity and mechanism) and variations in protocol adherence or tourniquet application could contribute to these findings. This emphasizes the importance of high-quality prospective studies to validate the utility of tourniquets in diverse operational contexts.

Given the notable difference in the Henry (2021) study [27], a sensitivity analysis was conducted without it; however, this did not change the outcome (RR 0.76, 95% CI 0.51–1.14).

#### 3.2.2. Delayed Amputation

**Figure 5:**
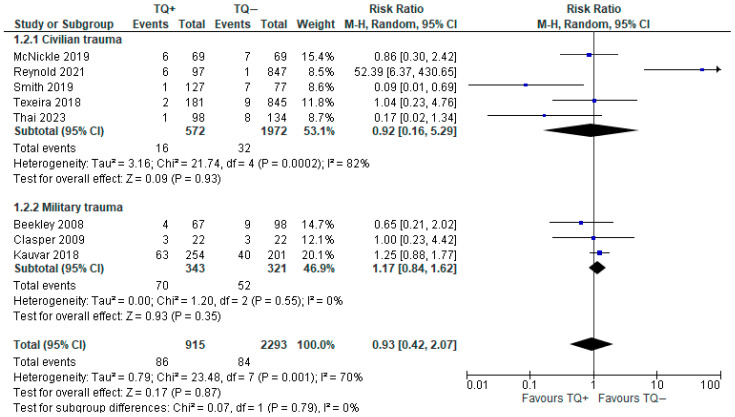
Risk ratio for delayed amputation with prehospital TQ (TQ+) vs. no prehospital TQ (TQ−) [11,26,27,28,29,30,31,34].

A total of eight observational studies (3208 patients) reported this outcome [11,26,27,28,29,30,31,34]. Despite differences among some of the included observational studies, the forest plot showed a higher incidence of amputation of the traumatized limb in the prehospital tourniquet group (9.39%) than in the no tourniquet group (3.66%); however, this result was not statistically significant (RR 0.93, 95% CI 0.42–2.07). Heterogeneity was high (I^2^ = 70%) (Figure 5).

In the subgroup analysis, we reported two different trends of delayed amputation in the TQ group: a lower rate in the civilian group (2.8%) vs. a higher rate in the military group (20.8%).

The risk ratio for delayed amputation remained non-significant after sensitivity analysis with the removal of Henry (2021) [27].

### 3.3. Secondary Outcomes

#### 3.3.1. Early Amputation

**Figure 6:**
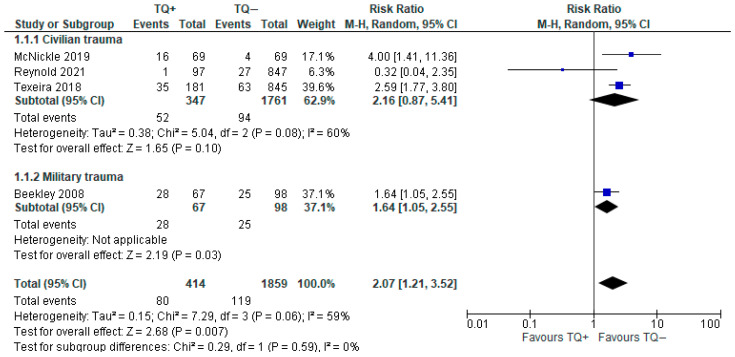
Risk ratio for initial amputation with prehospital TQ (TQ+) vs. no prehospital TQ (TQ−) [11,27,28,31].

A total of four observational studies (2273 patients) were included for this outcome [11,27,28,31]. The cause of amputation of the damaged limb was never reported. Despite differences among some of the included observational studies, the forest plot showed a higher frequency in the prehospital tourniquet group (19.32%) than in the no tourniquet group (6.4%), and the result was statistically significant (RR 2.07, 95% CI 1.21–3.52). Heterogeneity was found to be intermediate (I^2^ = 59%). The subgroup analysis reported the same trend in the overall result, but in the civilian subgroup, the result was not statistically significant for the conflicting data reported in the study of Henry [27] (Figure 6).

Furthermore, we performed a sensitivity analysis considered without the Henry (2021) study; the result was not different to the previous analysis: (RR 2.28, 95% CI 1.50–3.47).

#### 3.3.2. Transfused Blood Components

**Figure 7:**
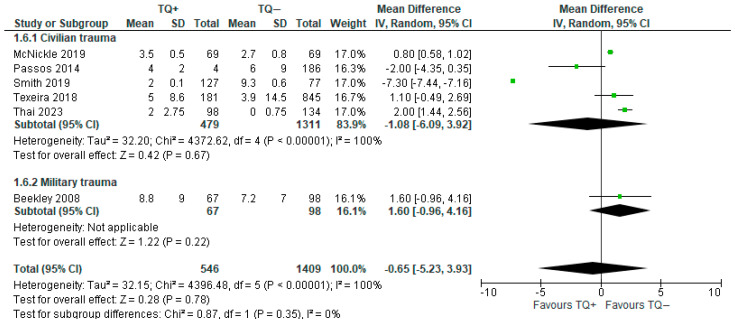
Mean difference in transfused pRBCs with prehospital TQ (TQ+) vs. no prehospital TQ (TQ−) [11,26,28,29,31,33].

Two types of blood components were analyzed:

Packed RBCs: six articles (including 1955 patients) showed benefits regarding tourniquet application [11,26,28,29,31,33]; in fact, the number of packed RBC units transfused was lower in the patients on whom a tourniquet was used, but the result was not statistically significant (MD = −0.65; 95% CI −5.23 to 3.93). Heterogeneity was very high (I^2^ = 100%). In the subgroup analysis, the transfusion of packed RBCs showed an opposite trend: a lower rate in civilian trauma patients that underwent tourniquet application vs. a lower rate in military trauma patients that did not undergo tourniquet application (Figure 7).

**Figure 8:**
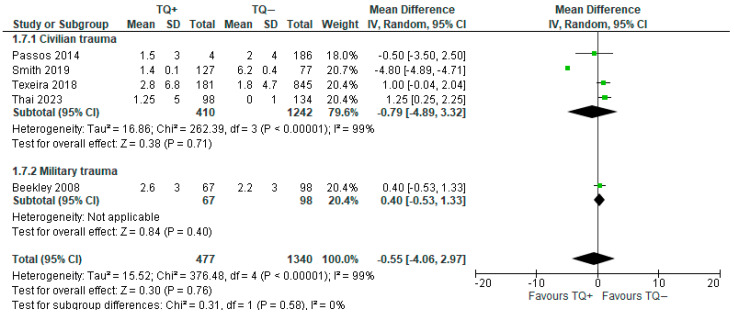
Mean difference in transfused plasma with prehospital TQ (TQ+) vs. no prehospital TQ (TQ−) [11,26,29,31,33].

Plasma: five articles (1817 patients) showed that tourniquet use reduced the number of plasma units transfused [11,26,29,31,33], but the result was not statistically significant (MD = −0.55; 95% CI −4.06 to 2.97). Heterogeneity was found to be very high (I^2^ = 99%). A similar trend of opposite results was reported in the subgroup analysis (Figure 8).

## 4. Discussion

The objective of this systematic review and meta-analysis was to evaluate the clinical effectiveness of prehospital tourniquets for controlling extremity bleeding, with a specific focus on mortality reduction, limb salvage, and transfusion requirements in both civilian and military settings. A total of 3686 patients (2790 for civilian trauma and 896 for military trauma), reported in 11 observational studies, were included in this meta-analysis, which demonstrated a significantly higher frequency of early amputations of the injured limb in the tourniquet group. However, there is insufficient evidence to state that this is solely related to tourniquet use, with additional factors such as initial injury severity also likely to be contributors. There were no other significant differences between patients receiving a prehospital tourniquet, compared to those without a tourniquet, in relation to mortality, late amputation rates, and transfusion. The subgroup analysis of civilian and military patients showed a higher rate of delayed amputations of the injured limb in the military group with tourniquet application. The opposite results for transfusion rate (lower in civilian group with tourniquet vs. lower in military group without tourniquet) is probably associated with a selection bias regarding the patients enrolled in the military group, which included only one study.

There are already two systematic reviews and meta-analyses in the literature, but they appear to be different from ours.

Latina et al. [35] conducted a meta-analysis of the clinical effectiveness of tourniquets in a prehospital setting. Their analysis, which included four studies, did not show a clear benefit of prehospital tourniquet application in terms of survival rates and the use of blood products. Although their results were not different from our results, our meta-analysis included 11 trials and had a larger number of participants (the total number of patients was 3686). In addition, we included observational studies conducted in military settings and performed a subgroup analysis to highlight differences in outcomes in civilian and military patients.

Ying-Chih Ko [36] et al. analyzed the effectiveness of prehospital tourniquets in a civilian patient population. Their study found that prehospital tourniquet use was associated with increased survival, while it was not associated with a decrease in the use of blood products. In this meta-analysis, seven studies were included. Patients with no tourniquet and patients with in-hospital tourniquet application were included in the control group. For comparison, in our study, the control group consisted only of patients who did not have a tourniquet applied. We excluded from our meta-analysis two studies by Ko et al. (Scerbo, Schroll), for the reasons mentioned above [36].

The results of the current meta-analysis may make it a new landmark study on which to base the eventual update of the guidelines published in 2014 by the American College of Surgeons Committee on Trauma and the Hartford Consensus Group. These guidelines recommend the use of a tourniquet when limb bleeding cannot be controlled with direct pressure and is life-threatening. This trend was successively supported by the Stop the Bleed campaign developed by the American College of Surgeons to raise public awareness, standardize training, and educate non-medical bystanders in early external bleeding control techniques [37].

### 4.1. Limitations

The included studies evaluated the results of the use of tourniquets in both civilian and military settings. The lack of randomized controlled trials, and the retrospective nature of >90% of the included studies, which may have led to bias in data collection and reporting, are limitations. The available literature is heterogeneous, with great variability in cohort composition, data collected, outcomes, and injury mechanisms. The characteristics of the injuries to which tourniquets were applied appeared to vary between studies, and only three studies [27,28,31] provided information on the vessels affected by the lesions. Because these were retrospective studies, with data obtained primarily from trauma registries and medical records, information on tourniquet duration was not always available, and when it was available, it showed wide variability between studies. Only two studies [29,32], indicated that most tourniquets were applied by healthcare providers, and few studies indicated the existence of a protocol for tourniquet application. There also appears to be a lack of information regarding the appropriateness and correct placement of tourniquets. It is also important to highlight a possible indication bias: tourniquets were more likely to be applied in patients with severe injuries and a higher risk of death. Future studies that focus on systematically collecting and reporting prehospital mortality data to better capture the impact of tourniquet application during the prehospital phase are necessary. Another limitation is the absence of detailed reporting on mortality timelines and causes of death.

### 4.2. Clinical Implications

While tourniquets are widely used in the military, their use in the civilian context is not yet widespread, although it is steadily growing. Hashmi’s study indicates that the estimated incidence of tourniquet use in the civilian setting is 1.2 per 1000 emergency medical service (EMS) activations [38], which is higher than the 0.2 per 1000 activations reported by El Sayed in 2016 [13]. The increase in tourniquet use suggests that the efforts of the American College of Surgeons Committee on Trauma and the Hartford Consensus, as well as the Stop the Bleed awareness campaign, have had an impact on prehospital bleeding management.

The results of the meta-analysis of the included studies indicate that there are insufficient statistically relevant data to determine the effectiveness of tourniquets in terms of survival, limb salvage, and transfusion requirements. Despite variability in study characteristics, we believe, in accordance with current guidelines, that tourniquets are a useful tool in controlling severe extremity bleeding when direct compression is ineffective. This is especially true in scenarios involving large numbers of people, such as mass shootings, terrorist attacks, natural disasters, and other situations in which the number of traumatized individuals may exceed the number of rescuers. In such scenarios, direct compression can be time-consuming and technically complex. The modern tourniquet, on the other hand, is a simple, lightweight, portable, inexpensive instrument that is easy to use and effective at controlling bleeding. With proper training, it can be used effectively by civilians to increase the chances of controlling bleeding until medical assistance arrives, potentially saving lives. Therefore, awareness and education campaigns, such as Stop the Bleed, are critical to the improvement of the management of life-threatening limb bleeding.

## 5. Conclusions

The effectiveness of tourniquets for reducing mortality, delayed amputation of the damaged limb, and blood product requirements is not well defined, according to our data. To validate the initial findings, further research on the use of tourniquets in a civilian setting is warranted.

## Figures and Tables

**Table 1:** General characteristics of the studies (TQ: tourniquets, NR: not reported, pRBCs: packed red blood cells, ROS: retrospective observational study, POS: prospective observational study, FFP: fresh frozen plasma, USA: United States of America).

Author, Year	Study	Setting	Duration	N. Partecipants	TQ+ Group	TQ− Group	NR	Mean Age	Male %	Outcomes
Thai 2023 [26]	ROS	USA	June 2016–May 2021	232	98	134	0	Years (IQR) TQ+ 32 (24–48) TQ− 33(25–44)	TQ+ 85.7 TQ− 84.3	Transfused pRBC and FFP Delayed amputation Mortality
Reynold 2021 [27]	ROS	USA	October 2015–July 2019	944	97	847	0	Years (SD) TQ+ 34.8 (13.3) TQ− 36.8 (12.4)	TQ+ 85.6 TQ− 84.1	Transfused pRBC Delayed amputation Mortality
McNickle 2019 [28]	ROS	USA	January 2013–December 2017	138	69	69	0	Years (SEM) TQ+ 35.0 (1.5) TQ− 36.3 (1.6)	TQ+ 81 TQ− 77	Delayed amputation Mortality
Smith 2019 [29]	ROS	USA	2010–2018	204	127	77	0	Years (SEM) TQ+ 31.3 (0.7) TQ− 31.2 (1.6)	TQ+ 87.4 TQ− 8.3	Transfused pRBC and FFP Delayed amputation and initial amputation Mortality
Kauvar 2018 [30]	ROS	USA	2004–2012	455	254	201	0	Years ± SD TQ+ 27 ± 7 TQ− 26 ± 6	NR	Delayed amputation Mortality
Teixeira 2018 [31]	ROS	USA	January 2011–December 2016	1.026	181	845	0	Years ± SD TQ+ 34.4 ± 14.7 TQ− 35.9 ± 13.8	TQ+ 87.2 TQ− 83.8	Transfused pRBC and FFP Delayed amputation and initial amputation Mortality
El Sayed 2017 [13]	ROS	USA	2011– 2014	10,366,537 EMS activations	2048	10,364,489	0	Years ± SD TQ+ 44 ± 21.1 TQ− 50.2 ± 25.8	TQ+ 76.5 TQ− 48.6	-
Ode 2015 [32]	ROS	USA	September 2012–November 2013	56	24	32	0	NR	NR	Mortality
Passos 2014 [33]	ROS	Canada	January 2001–December 2010	190	4	186	0	Years ± SD TQ+ 41 ± 12 TQ− 36 ± 16	TQ+ 100 TQ− 84	Transfused pRBC and FFP Mortality
Clasper 2009 [34]	ROS	Iraq. Afganistan	December 2003- May 2008	44	22	22	0	TQ+ 26.6 TQ− 25.7	NR	Delayed amputation
Kragh 2009 [9]	POS	Iraq	March 2006–October 2006	232	5	5	214	Years ± SD TQ+ 27 ± 5.5 TQ− 41 ± 11.5	TQ+ 100 TQ− 100	-
Beekly 2008 [11]	ROS	Iraq	January 2004–December 2004	165	67	98	0	TQ+ 28.5 TQ− 25	TQ+ 97 TQ− 96	Transfused pRBC and FFP Delayed amputation and initial amputation Mortality

## Data Availability

The data used to support the finding of this study are included within the article.

## Supplementary Materials

The following supporting information can be downloaded at: https://www.mdpi.com/article/10.3390/medicina61010093/s1, Table S1: Prehospital vital signs, Table S2: List of vascular injuries, Table S3: Mechanism of injury.
