# Supplementary material for: Is the Use of Tourniquets More Advantageous than Other Bleeding Control Techniques in Patients with Limb Hemorrhage? A Systematic Review and Meta-Analysis
Source: Medicina (Kaunas). 2025 Jan 9;61(1):93. doi: 10.3390/medicina61010093 (PMC11766969; doi:10.3390/medicina61010093)
Supplement: Supplementary file 1 [file medicina-61-00093-s001.zip › medicina-3366087-supplementary.pdf]

Supplementary Table S1 – Prehospital vital signs

Study	N		HR (bpm)		BP (mmHg)		GCS		ISS		Extremity AIS		MESS	
	TQ+	TQ-	TQ+	TQ-	TQ+	TQ-	TQ+	TQ-	TQ+	TQ-	TQ+	TQ-	TQ+	TQ-
**Thai 2023**	N = 232<br>98	134	-		-		-		Median (IQR)<br>10 (9-14.5)	10 (5-17)	-		-	
**Henry 2021**	N = 944<br>97	847	Mean (SD)<br>101 (18.3)<br>> 100 bpm, n (%)<br>43 (44.3)	96 (15.8)<br><br>313 (37.0)	Mean (SD)<br>113 (45.4)<br>< 90 mmHg, n (%)<br>17 (17.5)	119 (33.2)<br><br>122 (14.4)	Mean (SD)<br>14 (1.3)<br>< 9, n (%)<br>9 (9.3)	13 (2.5)<br><br>102 (12.0)	Mean (SD)<br>13.4 (8.1)	13.7 (7.3)	≥ 4, n, (%)<br>24 (24.7)	86 (10.2)	-	
**McNickle 2019**	N = 138<br>69	69	Mean (SEM)<br>110 (4)	100 (3)	Mean (SEM)<br>126 (4)	130 (4)	-		Mean (SEM)<br>13.1 (0.8)	12.3 (0.9)	Mean (SEM)<br>3.2 (0.1)	3.0 (0.1)	Mean (SEM)<br>5.8 (0.3)	5.1 (0.3)
**Smith 2018**	N = 204<br>127	77	Mean (SEM)<br>100 (2)	104 (5)	Mean (SEM)<br>114 (2)	98 (4)	-		Mean (SEM)<br>9.0 (0.5)	10.1 (0.6)	Mean (SEM)<br>2.8 (0.2)	2.7 (0.2)	-	
**Kauvar 2018**	N = 455<br>254	201	-		-		-		Mean ± SD<br>17 ± 9	16 ± 10	Median (IQR)<br>3 (3-4)	3 (3-3.5)	Median (IQR)<br>6 (5-7)	6 (5-7)
**Teixeira 2018**	N = 1,026<br>181	845	Mean ± SD<br>105.9 ± 28.7<br>> 100 bpm, n (%)<br>86 (51.8)	92.6 ± 27.4<br><br>287 (35.2)	Mean ± SD<br>125.3 ± 94.2<br>< 90 mmHg, n (%)<br>31 (18.9)	121.7 ± 34.3<br><br>106 (13.2)	≤ 8, n (%)<br>28 (15.7)	91 (10.9)	Mean ± SD<br>13.2 ± 10.3<br>≥ 16, n (%)<br>50 (27.6)	11.3 ± 8.4<br><br>179 (21.2)	≥ 4, n (%)<br>36 (20.0)	77 (9.1)	-	
**Ode 2015**	N = 56<br>24	32	-		-		-		> 15, n (%)<br>16 (69.9)	8 (25.8)	-		-	
**Passos 2014**	N = 190<br>4	186	Mean ± SD<br>99 ± 27	103 ± 26	Mean ± SD<br>99 ± 34	127 ± 30	Mean ± SD<br>11 ± 7	15 ± 1	Mean ± SD<br>17 ± 7	16 ± 10	-		-	
**Clasper 2009**	N = 44<br>22	22	-		-		-		Mean<br>10<br>> 15, n<br>10	10<br><br>9	-		Mean<br>5	5
**Kragh 2009**	N = 232<br>232		-				-		Mean ± SD<br>16	16	-		-	
**Beekley 2008**	N = 165<br>67	98	Mean ± SD<br>102 ± 35	101 ± 31	Mean ± SD<br>108 ± 36	108 ± 32	-		Mean ± SD<br>16.8 ± 14	17.5 ± 14	Mean ± SD<br>3.5 ± 1.1	3.4 ± 1.0	-	

N = number; HR = heart rate; BP = blood pressure; GCS = Glasgow coma score; ISS = Injury severity score; AIS = Abbreviated injury scale; MESS = Mengled extremity severity score; SD = Standard deviation; SEM = Standard error of mean; IQR = Interquartile range.

Supplementary Table S2 – List of vascular injuries

Vascular injuries	Henry 2021		McNickle 2019	Teixeira 2018	
	TQ+ (n = 97) n (%)	TQ- (n = 847) n (%)	TQ+ (n = 69) n (%)	TQ+ (n = 181) n (%)	TQ- (n = 845) n (%)
**Axillary artery**	1 (1.0)	29 (3.4)			
**Axillary vein**	1 (1.0)	13 (1.5)			
**Brachial artery**	16 (16.5)	102 (12.0)	20 (29)	40 (22.1)	151 (17.9)
**Brachial vein**	5 (5.2)	17 (2.0)			
**Radial/Ulnar artery**	39 (40.2)	212 (25.0)	23 (33)		
**Radial artery**				50 (27.6)	212 (25.1)
**Ulnar artery**				41 (22.7)	222 (26.3)
**Cephalic/Basilic vein**	11 (11.3)	39 (4.6)			
**Femoral artery**	6 (6.2)	136 (16.1)	6 (9)		
**Common femoral a.**				11 (6.1)	44 (5.2)
**Superficial femoral a.**				25 (13.8)	84 (9.9)
**Deep femoral a.**				9 (5.0)	18 (2.1)
**Femoral vein**	3 (3.1)	69 (8.2)		21 (11.6)	72 (8.5)
**Popliteal artery**	10 (10.3)	88 (10.4)	10 (14)	19 (10.5)	97 (11.5)
**Popliteal vein**	5 (5.2)	22 (2.6)		12 (6.6)	34 (4.0)
**Tibial/Peroneal artery**	7 (7.2)	148 (17.5)	10 (14)		
**Anterior tibial a.**				13 (7.2)	37 (4.4)
**Posterior tibial a.**				13 (7.2)	42 (5.0)
**Non specified tibial vessel**				13 (7.2)	29 (3.4)
**Anterior Tibial vein**				7 (3.9)	8 (0.9)
**Posterior Tibial vein**				8 (4.4)	12 (1.4)
**Saphenous vein**	3 (3.1)	23 (2.7)		10 (5.5)	26 (3.1)

Supplementary Table S3 – Mechanism of injury

**Mechanism of injury**	**Thai** TQ+ (n=98)	**Thai** TQ- (n=134)	**Henry** TQ+ (n=97)	**Henry** TQ- (n=847)	**McNickle** TQ+ (n=69)	**McNickle** TQ- (n=69)	**Smith** TQ+ (n=127)	**Smith** TQ- (n=77)	**Teixeira** TQ+ (n=181)	**Teixeira** TQ- (n=845)	**Ode** TQ+ (n=24)	**Ode** TQ- (n=32)	**Clasper** TQ+ (n=22)	**Clasper** TQ- (n=22)
**Penetrating Trauma n (%)**	83 (84.7)	96 (71.6)	49 (50.5)	288 (34.0)										
**Gunshot wounds n (%)**					10 (14)	15 (22)	54 (42.5)	50 (64.9)			4 (16.7)	11 (34.4)		
**Knife, glass, saw, other penetrating objects wounds n (%)**					Knife<br>12 (17)<br>Glass, saw, other penetrating objects<br>17 (25)	11 (16)<br><br>10 (14)	Knife, saw<br>30 (23.6)<br>Glass, other penetrating objects<br>41 (32.3)	14 (18.2)<br><br>13 (16.9)			Knife<br>2 (8.3)	5 (15.6)		
**Bite n (%)**					1 (1)	2 (3)	2 (1.6)	0						
**Blunt trauma n (%)**	14 (14.3)	37 (27.6)							81 (44.8)	342 (40.5)				
**Burn n (%)**	1 (1.0)	1 (0.8)												
**Motor vehicle collision n (%)**					11 (16)<br>Motorcycles, off-road vehicles<br>12 (17)	9 (13)<br><br>15 (22)					5 (20.8)	2 (6.3)		
**Pedestrian/ fall n (%)**					6 (9)	7 (10)					Fall<br>1 (4.2)	1 (3.1)		
**Explosions n (%)**													7 (31.8)	14 (63.6)
**Machinery injury n (%)**											2 (8.3)	1 (3.1)		
**Arteriovenous fistula rupture n (%)**											4 (16.7)	4 (12.5)		
**Traumatic laceration n (%)**											5 (20.8)	8 (25)		
**Varicose vein hemorrhage n (%)**											1 (4.2)	1 (3.1)		

Specific search strategies for each database:

- PubMed

"tourniquet and trauma and prehospital"

- Scopus

"tourniquet AND trauma AND prehospital"

- Web of Science (WOS).

"tourniquet AND trauma AND prehospital"
